# Performance Evaluation of a New Coulometric Endpoint Method in Sweat Testing and Its Comparison With Classic Gibson&Cooke and Chloridometer Methods in Cystic Fibrosis

**DOI:** 10.3389/fped.2018.00133

**Published:** 2018-05-22

**Authors:** Yasemin Gokdemir, Pinar Vatansever, Bulent Karadag, Tuncay Seyrekel, Ozgur Baykan, Nilay Bas Ikızoglu, Refika Ersu, Fazilet Karakoc, Goncagul Haklar

**Affiliations:** ^1^Department of Pediatric Pulmonology, School of Medicine, Marmara University, Istanbul, Turkey; ^2^Department of Biochemistry, School of Medicine, Marmara University, Istanbul, Turkey; ^3^Biochemistry Laboratory, Yozgat City Hospital, Yozgat, Turkey; ^4^Biochemistry Laboratory, Ataturk State Hospital, Balikesir, Turkey

**Keywords:** sweat test, cystic fibrosis, coulometric endpoint system, diagnosis, chloride

## Abstract

**Background:** The objective of the study was to assess the diagnostic efficacy of the coulometric endpoint method and compare it with classic Gibson&Cooke and chloridometer methods.

**Methods:** This study is a prospective clinical study comparing two conventional sweat testing methods with the coulometric endpoint method in previously diagnosed cystic fibrosis (CF) patients and a non-CF control group. All individuals underwent two simultaneous sweat collections. One sample of sweat, collected by the CFΔ collector coil system, was analyzed by two methods: the titrimetric Cl^−^ measurement (Sherwood® Chloridometer 926S, Sherwood Scientific Ltd., Cambridge, UK) and the coulometric endpoint method (CF Δ Collection System®, UTSAT/Turkey); the second sample was collected from the other forearm by the Gibson&Cooke method and the collected sweat was analyzed by manual titration in accordance with the Schales&Schales method. Within-run and between-run imprecisions were evaluated via Cl^−^ concentrations of 40, 70, and 130 mmol/L samples.

**Results:** One hundred and seventy (60 CF and 110 controls) subjects were included in the study.

All three sweat test methods discriminated CF subjects from the healthy individuals. The mean difference between the coulometric endpoint and titrimetric Cl^−^ measurement methods was −1.5 mmol/L, (95% confidence limits of agreement, ranging from −8.9 to 15.9 mmol/L); the mean difference between manual titration vs. coulometric endpoint methods was 12.8 mmol/L, (95% confidence limits of agreement ranging from −9.7 to 45.3 mmol/L) and the mean difference between the manual titration and titrimetric Cl^−^ measurement methods was 11.3 mmol/L, (95% confidence limits of agreement ranging from −7.8 to 40.5 mmol/L) based on a Bland-Altman analysis. In the Receiver operating characteristic (ROC) analysis, made on the basis that Cl^−^ concentration values < 40 mmol/L exclude the CF diagnosis, the coulometric endpoint method resulted in 96.7% sensitivity and 100% specificity for a cut-off value of 58.5 mmol/L (AUC: 0.994; 95% CI = 0.986–1.000; *p* < 0.001).

**Conclusions:** The coulometric endpoint method can be as reliable as quantitative sweat Cl^−^ analysis and may be considered as a definitive diagnostic tool for CF.

## Introduction

Cystic fibrosis (CF) diagnosis is based on neonatal screening findings and/or phenotypic manifestations, together with family history, and confirmed by high chloride ion (Cl–) concentration in sweat (1–10). Currently, the sweat chloride concentration level has also been useful to demonstrate the function of the CFTR protein after the administration of corrector, potentiator, or stabilizer drugs. Therefore, besides diagnosing CF, the future role of sweat test may include the successful monitoring of personalized medicine therapy (4–6).

The Gibson&Cooke quantitative pilocarpine iontophoresis test (QPIT), measuring sweat Cl^−^ concentration, is accepted as the standard sweat test method for the diagnosis of cystic fibrosis. However, this conventional procedure carries a significant risk of failure unless carried out by trained, experienced personnel; errors made during collection and analysis can lead to volumetric, gravimetric, condensate, and evaporation inaccuracies (1–10).

In the past Macroduct® coils (Wescor, Logan, UT) for sweat collection have been commonly used in many CF centers, simplifying the employment of QPIT (5). The Chloridometer which is a conventional instrument for Cl^−^ concentration analysis, also utilizes the Macroduct® sweat collection system (6).

The sweat coulometry system is simpler and does not involve the steps of weighing and dilution; it also reduces the risk of sample evaporation since sweat is collected via a CFΔ collector system similar to Macroduct® coils. The coulometric endpoint tecnique is an analytical chemistry technique that utilizes an electrolysis reaction to measure the changes in resistance to the current between electrodes; the concentration of the titrant is equivalent to the current generated. This method is approved by the Clinical Laboratory Standards Institute (CLSI) United Kingdom and Australian sweat test guidelines (3–5). The CFΔ Collection System® is a new generation sweat test analyser manufactured by Utsat, from Turkey, based on the coulometric endpoint method.

In this prospective study, we performed sweat tests in patients with previously diagnosed CF and in the non-CF control group. Three sweat test analyses were carried out for each patient, simultaneously, by manual titration, titrimetric Cl^−^ measurement by chloridometer (Sherwood® Chloridometer 926S, Sherwood Scientific Ltd., Cambridge, UK), and the coulometric endpoint method (CF Δ Collection System®, UTSAT/Turkey).

The aim of our study was comparison of diagnostic accuracy for sweat testing between a new technique by coulometric endpoint method and conventional methods (Gibson&Cooke and chloridometer).

## Methods

This study was carried out at the Pediatric Pulmonology Department and Biochemistry Laboratory of Marmara University of Medicine between September 16 and October 1, 2013. The study was approved by the ethical committee of Marmara University of Medicine with a grant from TUBITAK 1507 R&D projects.

One hundred and seventy (60 CF and 110 controls) subjects were included in the study. CF patients with clinical findings and laboratory evidence of CFTR dysfunction and with elevated sweat Cl^−^ concentrations on at least two tests, and/or presence of two CF causing mutations, were recruited. Patients were excluded if they were younger than 2 weeks of age, had edematous extremities or signs of dehydration, or had a body weight of < 3 kg. The control group consisted of healthy individuals known to have normal sweat Cl^−^ values who were first degree relatives of the CF patients. The exclusion criteria for the control group were presence of edematous extremities, hypothermia, or signs of dehydration and use of corticosteroids.

All individuals underwent two simultaneous sweat collections. One sample of sweat was collected using the CFΔ collector coil system, with the sweat analyzed by two methods: titrimetric Cl^−^ measurement (Sherwood® Chloridometer 926S, Sherwood Scientific Ltd., Cambridge, UK) and the coulometric endpoint method (CF Δ Collection System®, UTSAT/Turkey). The second sample was collected from the other forearm by the Gibson&Cooke method and the collected sweat was analyzed only by manual titration, using the Schales & Schales method. Conventional sweat tests were performed in accordance with the standards of the CLSI and by the same qualified technician (3).

The CF Δ Collection System® (UCF 2010 Iontophoresis Unit and UCF 2011 Sweat Analysis Unit) analyzes Cl^−^ concentration of sweat with the coulometric endpoint software method.

In the coulometric endpoint and chloridometer methods, iontophoretic stimulation of sweat glands was done by placing two electrodes on the forearm on which were placed discs of pilocarpine nitrate gel. Maximum 1.5 mA current was applied to these electrodes in 5–7.5 min time. For sweat-collection, CFΔ collector was used; it is a disposable, concave, plastic disc with a hole in the center attached to a spiral plastic tube inside. This spiral tube has a total capacity of 100 μL. Sweat was collected during a period of 30 min and Cl^−^ concentration was analyzed with both the Sherwood® Chloridometer 926 S Analyser and the CFΔ Collection System® Analyser.

With regards to the analysis, the CF Δ Collection System® uses a coulometric endpoint software for the measurement of sweat Cl^−^ concentration. With this device it is possible to measure both the conductivity and the Cl^−^ concentration of sweat at the same time, using just 4.1–6 μL of sweat. It has a peristaltic pump that transfers sweat directly from the microbore tubing which means there is no need for manual pippetting. This method measures the number of electrons flowing through the sweat sample by applying potential difference on two electrodes in microvolumed and constant temperature-controlled measurement cells. It is then processed in microvolumed measurement cells. The results are defined in mmol/L Cl^−^ content and compatible NaCl^−^ value. Sweat conductivity and sweat Cl^−^ concentration are measured simultaneously and the results appear on the digital display.

The interpretation of sweat test results was in accordance with the United States Cystic Fibrosis Foundation guidelines (7). For subjects ≥ 6 months of age, a sweat Cl^−^ of ≤ 39 mmol/L was normal, 40–59 was intermediate, and ≥ 60 mmol/L was abnormal and consistent with CF.

Within-run and between-run imprecisions were determined using standards with 40, 70, and 130 mmol/L Cl^−^ concentrations, in accordance with the “Evaluation of Precision Performance of Quantitative Measurement Methods; Approved Guideline (EP5-A2) of CLSI.” Bias was determined from the % difference in the serial measurements of standard materials from their indicated value. Methods were compared as per “Method Comparison and Bias Estimation Using Patient Samples; Approved Guideline (EP9-A2) of CLSI (3).”

### Statistical analysis

Statistical analyses were carried out using the SPSS 15.0 and MedCalc 13.0 software. For all analyses *p* < 0.05 was used to consider statistical significance. Bland-Altman plots were used to compare the results of the two methods. As a measure of agreement on the results of the methods, Passing-Bablok regression analysis was performed to determine if the residuals were randomly distributed around the regression line. The sensitivity and specificity of the sweat test values were determined with receiver operating characteristic (ROC) curve.

## Results

**Figure 1:**
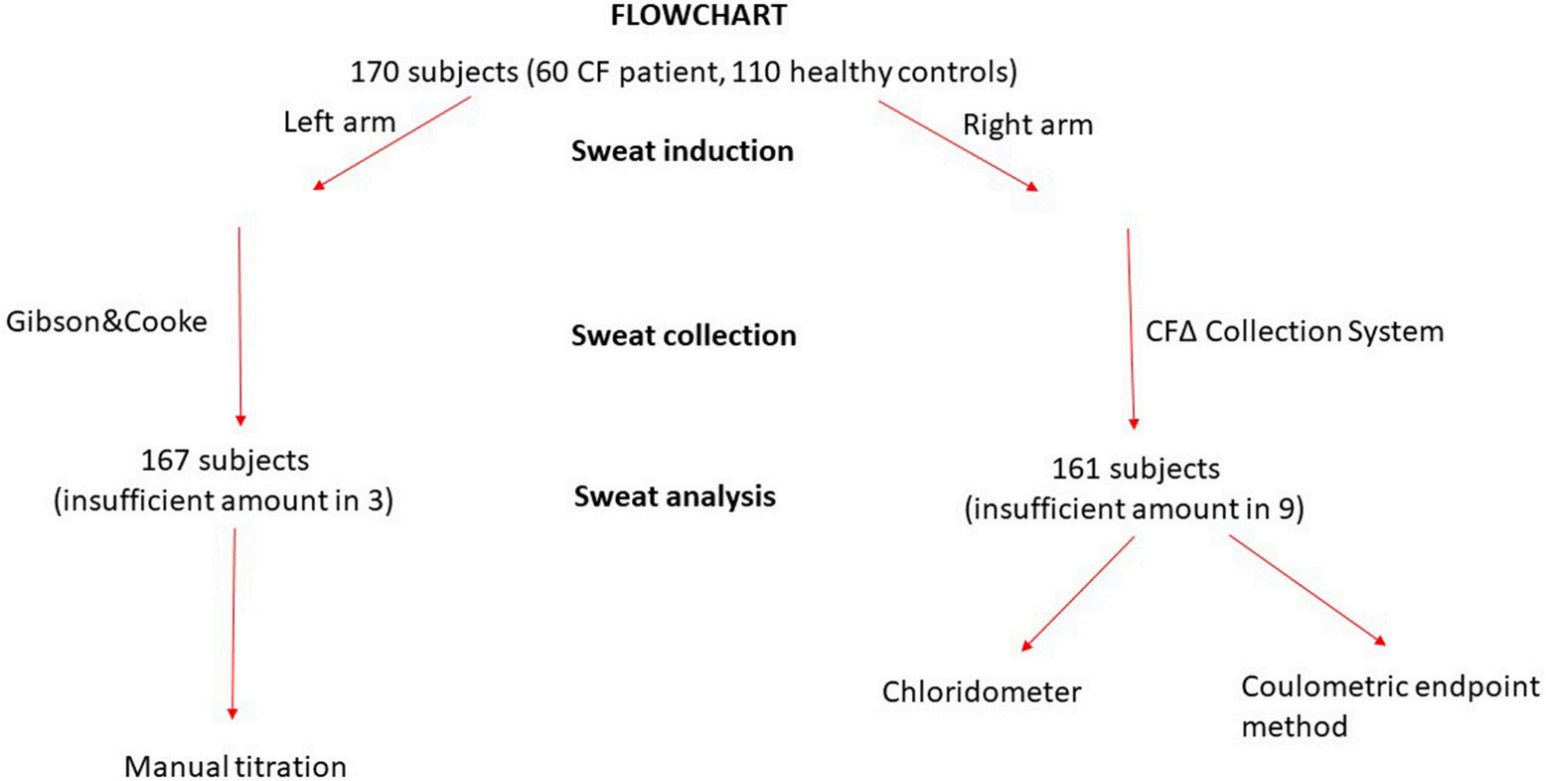
Flowchart of the study.

One hundred and seventy (60 CF and 110 controls) subjects were included in the study. Iontophoretic stimulation of sweat glands was carried out in 170 subjects and adequate sweat was collected in 161 of them. The volume of sweat collected using the CFΔ collector system was insufficient in 9 (5.3%) subjects, and was also insufficient in 3 subjects, where sweat was collected from the other arm using the Gibson &Cooke method. No adverse events happened during sweat testing procedures (Figure 1).

50.7% of the CF group were male, and 43% of the control group (*p* = 0.303) were male. The mean age of the CF group was 9.0 ± 6.1 (1–21) and for the control group it was 21.7 ± 16.6 (1–58) (*p* < 0.001) years old. In the CF subjects sweat test results were ≥ 60 mmol/L with all three of the methods employed.

Within-run and between-run imprecision data are summarized in Table 1. As seen in this table the lowest within-run and between-run imprecision values were obtained by coulometric measurement. Bias assessments were performed with the 40, 70, 130 mmol/L standards and the coulometric measurements were the most accurate (Table 2).

**Table 1:** Precision studies of the assays with 40, 70, 130 mmol/L standarts.

	**Manual titration**			**Chloridometer**			**Coulometric endpoint method**		
Standard concentration (mmol/L)	40	70	130	40	70	130	40	70	130
Within-run CV(%)	10.3	8.9	10.8	7.57	6.77	6.41	0.54	0.21	0.47
Between-run CV (%)	4.19	6.01	5.50	6.16	6.78	7.06	2.72	2.13	1.08

**Table 2:** Bias studies of the assays with 40, 70, 130 mmol/L standards.

	**40 mmol/L**		**70 mmol/L**		**130 mmol/L**	
	**Mean (mmol/L)**	**Bias (%)**	**Mean (mmol/L)**	**Bias (%)**	**Mean (mmol/L)**	**Bias (%)**
Manual titration	35.78	10.6	85.42	22	144.2	10.9
Chloridometer	30.91	23	68.67	1.9	138.5	1.1
Coulometric endpoint method	38.97	2.6	67.28	3.9	130.1	0.07

**Figure 2:**
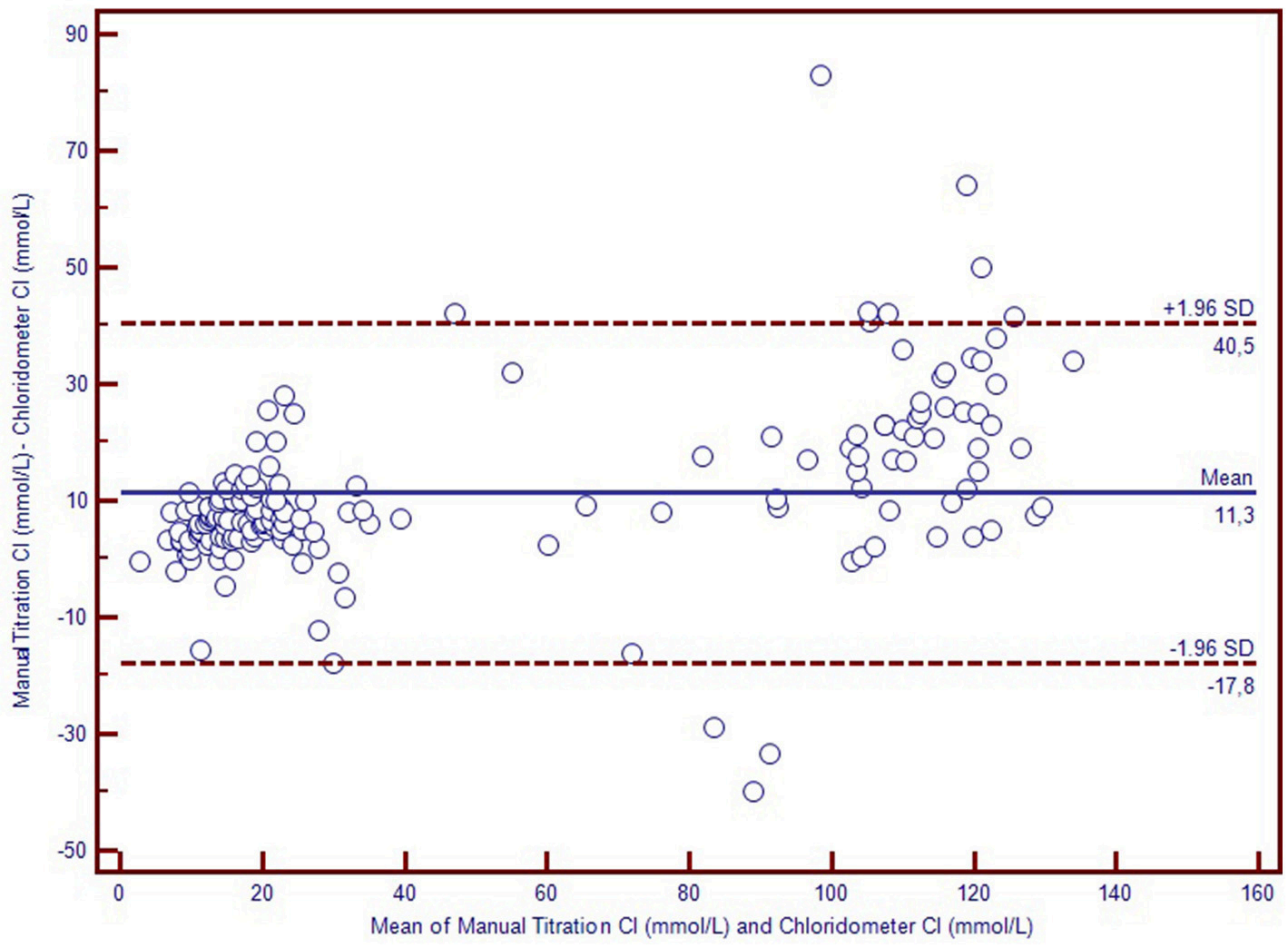
Bland-Altman Plot of manual titration vs. chloridometer.

**Figure 3:**
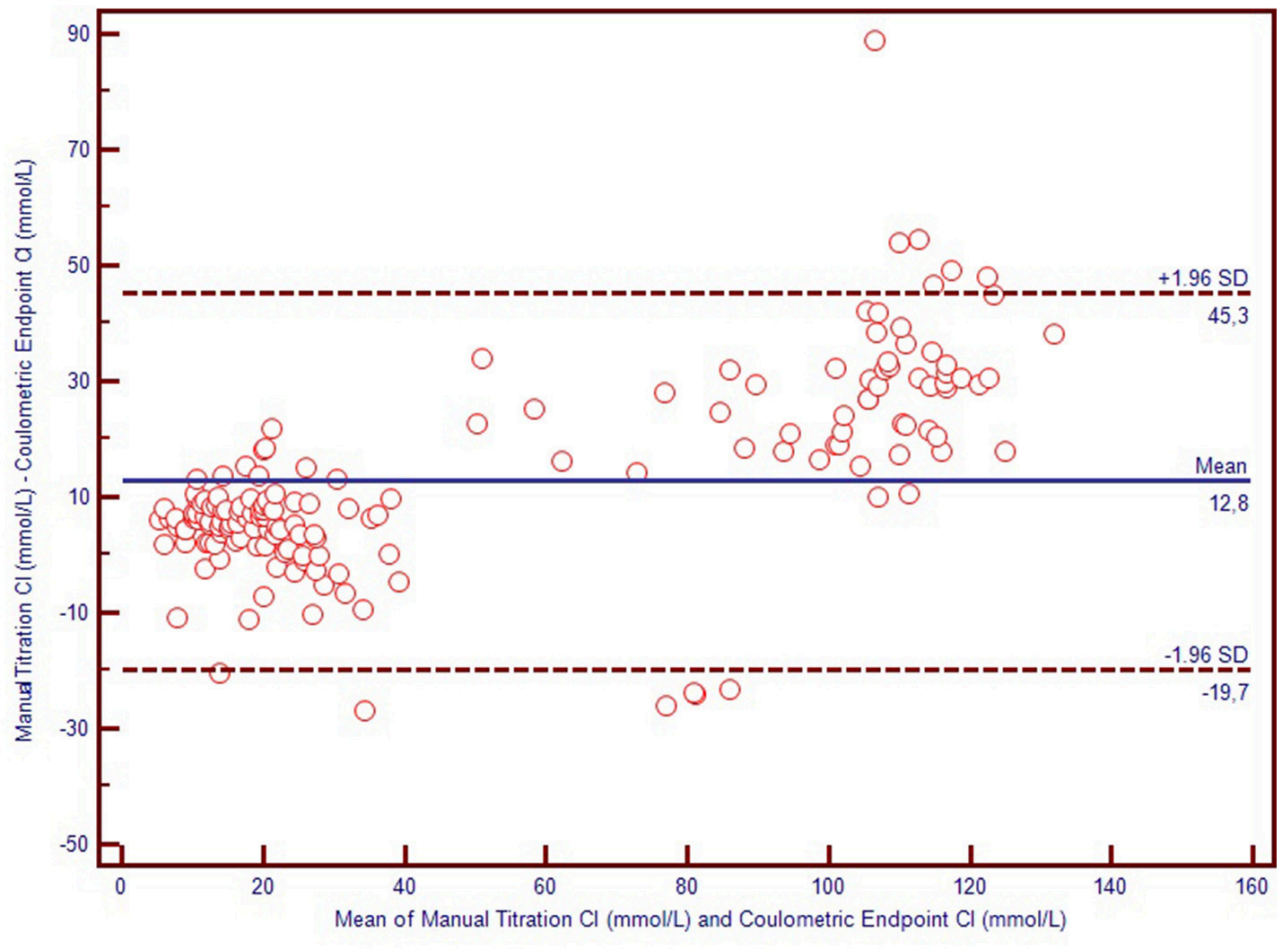
Bland-Altman Plot of manual titration vs. coulometric endpoint method.

**Figure 4:**
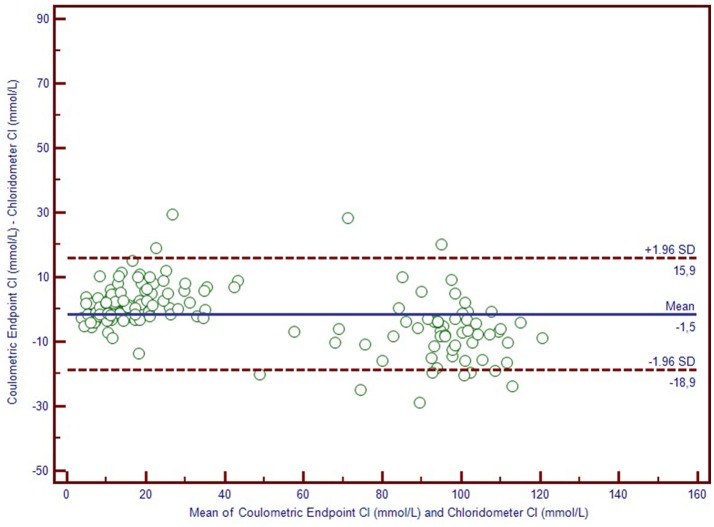
Bland-Altman Plot of chloridometer vs. coulometric endpoint method.

We used Bland-Altman plots (difference plots) to analyse the agreement between methods in the healthy controls and the CF subjects. We visually observed a systematic bias between manual methods and Sherwood® chloridometer in the Bland-Altman plot constructed by mean to difference measurements, where the discrepancy increased as the concentations increased (Figure 2). Although the same bias was observed in the Bland-Altman plot of manual titration vs. the coulometric endpoint method (CF Δ System®), the discrepancy among the healthy population was less prominent (Figure 3). The results of the Sherwood® chloridometer and the coulometric endpoint methods (CF Δ System®) were more in agreement and resulted in a well-matched Bland-Altman plot (Figure 4).

The agreement of the methods was tested with Passing and Bablok analysis; this showed that in CF patients manual titration methods had both systematic and proportional difference when compared with chloridometer and coulometric endpoint methods, where chloridometer, and coulometric endpoint methods were more in agreement. None of the three methods showed any significant deviation from linearity (Table 3).

**Table 3:** The Passing Bablok regression analysis of the results of manual titration, Sherwood chloridometer, and coulometric end point methods (CF Δ System) in CF patients (*n* = 59).

**Method**	**Intercept**	**95% CI**	**Slope**	**95% CI**	**Deviation from linearity**
Manual titration-Sherwood chloridometer	17.26	−7.9–37.7	0.68	0.51–0.89	*P* = 0.59
Manual titration-CF Δ system	8.6	−12.8–29.5	0.7	0.53–0.87	*P* = 0.24
CF Δ system-Sherwood chloridometer	−3.56	−21.7–13.9	1.1	0.93–1.32	*P* = 0.78

**Figure 5:**
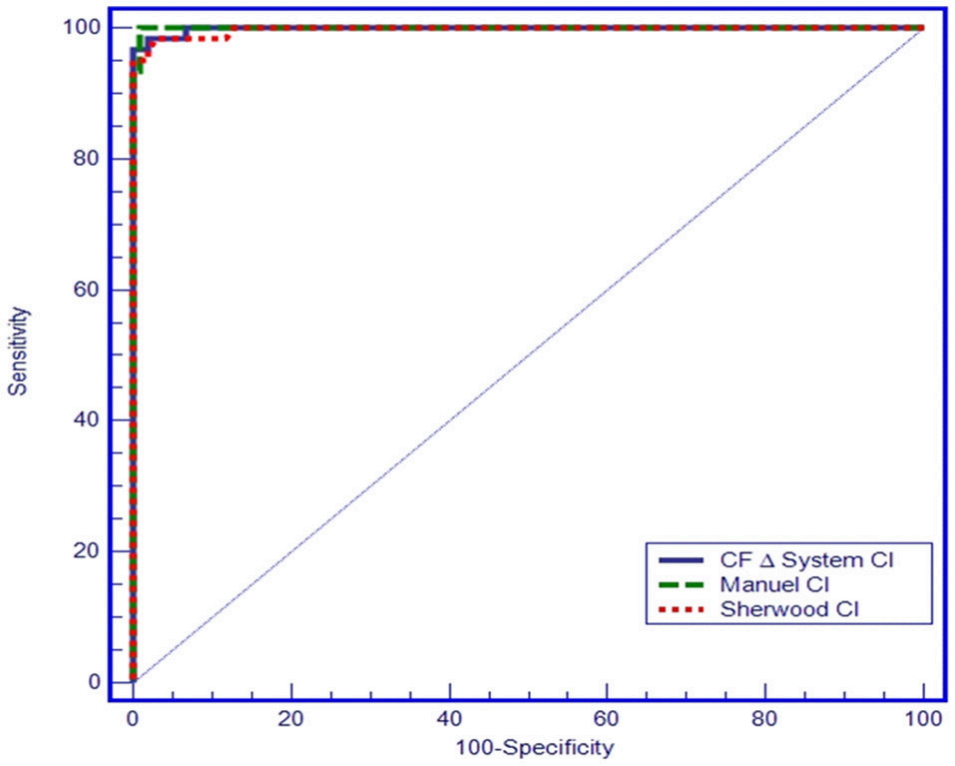
ROC curve of the three sweat test methods.

In the ROC analysis plotted on the basis that Cl^−^ concentration values < 40 mmol/L exclude a CF diagnosis, manual titration measurements resulted in 100% sensitivity and 99.02% specificity for a cut-off value of 61.5 mmol/L (AUC = 0.999, 95% CI = 0.976–1.000, *p* < 0.001), and chloridometer measurements resulted in 95% sensitivity and 100% specificity for a cut-off value of 60 mmol/L (AUC: 0.997; 95% CI = 0.972–1.000, *p* < 0.001). Likewise, the coulometric endpoint method (CF Δ System®) resulted in 96.7% sensitivity and 100% specificity for a cut-off value of 58.5 mmol/L (AUC: 0.997, 95% CI = 0.975–1.000, *p* < 0.001) (Figure 5).

## Discussion

This study compared a new sweat test method (coulometric endpoint method, CFΔ Collection System®, UTSAT/Turkey) with the Gibson&Cooke and titrimetric Cl^−^ measurement methods and has shown comparable sensitivity and specifity among the methods. It was demonstrated that this method reliably distinguishes normal (non-CF) subjects from persons with CF and has the potential for use as a new diagnostic tool for CF.

The Gibson&Cooke method has been considered as the gold standard for the diagnosis of CF since 1959 (1–6). This test, however, is very difficult to carry out. The whole procedure is vulnerable to error if not performed by experienced professionals who are specifically trained in sweat collection and analysis (1–6).

In past years the use of Macroduct® coils to facilitate sweat collection and make QPIT easier has become common in many CF centers (11–15). The CFΔ collector coil system, which is similar to the Macroduct® coil system, was used for sweat collection in this study; sample evaporation, therefore, is not a concern for this method.

With the CFΔ collector system the volume of sweat was insufficient in nine subjects, and with the Gibson &Cooke method on the other forearm, likewise, the volume collected was insufficient in three subjects. The CFΔ collector coil was used to collect sweat for two analyses at the same time; titrimetric Cl^−^ measurement and coulometric endpoint methods; this may be the reason that a sufficient volume could not be obtained via the CFΔ collector coil.

The need for standardization in the collection, analysis and reporting of sweat test results was addressed by the CLSI guideline in 1994 (14). According to the revised CLSI guideline, each CF center must perform quantitative pilocarpine iontophoresisis for sweat collection using either the Gibson&Cooke technique (GCT) or the Macroduct® coil system (3). Once collected, the sweat samples are then quantitatively analyzed for Cl^−^ concentration using a chloridometer, by manual titration, or via a previously validated automated analyzer, in order to obtain a result that meets clinical use standards (8).

Coulometry is described in the CLSI guidelines as an approved sweat test method. It is an analytical chemistry technique that utilizes an electrolysis reaction to measure the changes in resistance to the current between electrodes. The concentration of the titrant is equivalent to the current generated. The CF Δ System® (UTSAT/Turkey) is based on the coulometric endpoint method (6).

Although the average sweat collection time in coulometric methods is similar to that in conventional sweat testing, this device needs a lower volume of sweat (4.1–6 μL) than the Sherwood® chloridometer and other manual methods, which is very important when testing is carried out on newborns and infants. Another advantage of this device is that it has a peristaltic pump that transfers the sweat sample directly from the microbore tubing, meaning that manual pipetting, as with the Sherwood® chloridometer, is not required. This new peristaltic pump technique was also utilized in a new FDA approved chloridometer (the Elitech Chlorochek® Chloridometer).

Although there were differences between the sweat test results obtained with the three techniques employed in our study, all of them differentiated the subjects with and without CF. The manual method yielded sweat test results that were higher than those obtained with the chloridometer and coulometric end point methods, and this was related to the manual process employed in collecting and analyzing the sample. Sample evaporation is potentially a major pre-analytical error, as it can lead to false positive results when using the Gibson & Cooke method. The influence of the technician on results seems to play a greater role if the method is manually processed (3, 16, 17). In developing and undeveloped countries it is also difficult to carry out sweat testing using the Gibson&Cooke method due to a lack of experienced and well-trained technicians.

The sweat test has been reported to have high false-positive (up to 15%) and false-negative (up to 12%) results, due to inaccurate methodology, technical error, and patient physiology (18–21). There is also considerable intra-individual variation in sweat Cl^−^ levels of healthy individuals (21). This biological variation contributes to the total difference in sweat test results. A study that involved sweat testing of four healthy adults by conductivity on multiple occasions over a 2-year period demonstrated intra-individual biological variation for sweat Cl^−^ ranging from 14.2 to 32.8%. Another investigation of sweat Cl^−^ variation studied the difference between simultaneous sweat collections from the right and left arm. This study used the Gibson&Cooke QPIT method to compare 295 paired sweat tests; the results generated a coefficient of variation (CV) of 20.2% for Cl^−^ ion concentration (22).

Nguyen-Khoa et al. (23) compared sweat tests carried out using manual titration, chloridometer and conductivity; Cl^−^ ion concentrations were determined, with all three methods, in five different hospitals in CF patients and control standard samples, with Cl^−^ concentrations of 30, 50, and 100 mmol/L. Although intra-laboratory CVs were < 5% for values between 10 and 100 mmol/L, median inter-laboratory CVs were 9.0 (5.2–11.9) and 6.6 (3.4–9.0) in manual titration and chloridometer methods respectively, indicating a probable influence of the technicians carrying out the tests.

In our study, within-run and between-run imprecisions were determined using commercially available control samples with Cl^−^ concentrations of 40, 70, and 130 mmol/L. The lowest within-run and between-run imprecision values were obtained in coulometric endpoint measurements. Bias assessments were made with the same standards, and the coulometric endpoint system gave the most accurate results. According to the accuracy and precision studies, it was shown that manual methods were more vulnerable to user error than other methods. Differences between the results obtained from studies using manual methods were related to user error. Within-run and between-run VCs of coulometric endpoint systems remained < 5%, as prescribed by the UK guidelines (5, 24, 25).

Domingos et al. (26) compared the conductivity test with the quantitative coulometric test in suspected CF infants that had pathologic newborn screening tests. The conductivity test showed excellent correlation with the quantitative coulometric test, and high sensitivity and specificity, and the authors reported that it can be used in the diagnosis of CF in children detected through newborn screening. Although this study compaired the coulometric endpoint method with the other methods, within-run and between-run imprecisions were not performed.

To the best of our knowledge our study is the first to evaluate the within-run and between-run imprecisions of the coulometric endpoint sweat analysis technique.

One important limitation of this study was the study population selection; previously diagnosed CF patients were included in this prospective study. They all had classic CF related symptoms and high sweat Cl^−^ levels and the control group consisted of the healthy parents and siblings of the patients, and we did not carry out sweat tests on patients from the general population with respiratory symptoms suggesting CF. Consequently, the accuracy of this technique was not tested in patients with intermediate sweat test results.

The other limitation of our study was that sweat tests were not performed in newborns and infants with positive CF newborn screening test results, which is usually more cumbersome (4); the youngest patient in our study was 1 year old.

In conclusion, this is the first study to compare the coulometric endpoint sweat test method with other gold standard methods in CF and non-CF control groups, while evaluating precision and accuracy of the tests by performing within-run and between-run imprecisions. This new device using the CLSI approved coulometric endpoint method is easy to perform and requires a lower volume of sweat sample. It was found to be compatible with standard sweat test methods based on the Cl^−^ measurement and therefore shows promise as a diagnostic tool in the diagnosis of CF.

## Ethics statement

This study was carried out in accordance with the recommendations of Marmara Medical University guidelines, with written informed consent from all subjects. All subjects gave written informed consent in accordance with the Declaration of Helsinki. The protocol was approved by the ethical committee of Marmara University of Medicine.

## Author contributions

All authors listed have made a substantial, direct and intellectual contribution to the work, and approved it for publication.

### Conflict of interest statement

The authors declare that the research was conducted in the absence of any commercial or financial relationships that could be construed as a potential conflict of interest.

## Abbreviations

CF: Cystic fibrosis
QPIT: Quantitative pilocarpine iontophoresis test
CLSI: US Clinical Laboratory Standards Institute
Cl^−^: Chloride.
